# The political economy of farmers’ suicides in India: indebted cash-crop farmers with marginal landholdings explain state-level variation in suicide rates

**DOI:** 10.1186/1744-8603-10-16

**Published:** 2014-03-26

**Authors:** Jonathan Kennedy, Lawrence King

**Affiliations:** 1 Department of Sociology, University of Cambridge, Free School Lane, Cambridge, CB2 3RQ, UK; 2 Department of Political Science, University College London, London, UK

**Keywords:** Farmers’ suicides, Agrarian crisis, India, Marginal farmers, Cash crops, Indebtedness

## Abstract

**Background:**

A recent *Lancet* article reported the first reliable estimates of suicide rates in India. National-level suicide rates are among the highest in the world, but suicide rates vary sharply between states and the causes of these differences are disputed. We test whether differences in the structure of agricultural production explain inter-state variation in suicides rates. This hypothesis is supported by a large number of qualitative studies, which argue that the liberalization of the agricultural sector in the early-1990s led to an agrarian crisis and that consequently farmers with certain socioeconomic characteristics–cash crops cultivators, with marginal landholdings, and debts–are at particular risk of committing suicide. The recent *Lancet* study, however, contends that there is no evidence to support this hypothesis.

**Methods:**

We report scatter diagrams and linear regression models that combine the new state-level suicide rate estimates and the proportion of marginal farmers, cash crop cultivation, and indebted farmers.

**Results:**

When we include all variables in the regression equation there is a significant positive relationship between the percentage of marginal farmers, cash crop production, and indebted farmers, and suicide rates. This model accounts for almost 75% of inter-state variation in suicide rates. If the proportion of marginal farmers, cash crops, or indebted farmers were reduced by 1%, the suicide rate–suicides per 100,000 per year–would fall by 0 · 437, 0 · 518 or 0 · 549 respectively, when all other variables are held constant.

**Conclusions:**

Even if the Indian state is unable to enact land reforms due to the power of local elites, interventions to stabilize the price of cash crops and relieve indebted farmers may be effective at reducing suicide rates.

## Background

The *Lancet* recently published an article by Vikram Patel and his collaborators that used a nationally representative mortality survey of 1 · 1 million households to provide the first accurate estimates of deaths from suicide in India [1]. This important study revealed the magnitude of suicide as a public health problem. In 2010 187,000 people died from suicide in India–this amounts to one fifth of all suicides in the world [1,2]. Indian suicide rates–26 · 3 for men and 17 · 5 for women–are among the highest in the world [1-3]. Suicide is the second leading cause of death among young adults in India–after road accidents for men and maternity-related complications for women [1].

Patel et al.’s study demonstrated that suicide in India is a very different social phenomenon to suicide in high-income countries (HICs): suicide rates in rural areas are almost double those in urban areas, whereas in HICs there is little difference; suicide rates in India are highest in wealthier regions, which challenges findings in HICs; the most common method of suicide in India–the ingestion of pesticides–is rarely seen in HICs; and unlike elsewhere in the world, suicide rates in India are not higher among elderly people [2]. Consequently, as India undergoes the epidemiological transition and starts to turn its attention from infectious diseases to issues such as suicide prevention it cannot rely on approaches that have been successful elsewhere.

Patel et al. show that there is substantial geographical variation in suicides rates within India [1]. Suicide rates in some states are more than ten times those in others. Kerala, which is often presented as a model of success in terms of public health and human development, has the highest male suicide rate [4]. Indeed, if Kerala was a country its male suicide rate (66 · 3) would be the highest in the world–a position currently held by Lithuania (61 · 3) [1,5]. Bihar, on the other hand, which is one of the least developed states in India, has the lowest male suicide rate (6 · 3). This inter-state variation is a puzzle that has yet to be resolved by public health researchers. But it also provides us with an opportunity because, if we can understand why these differences occur, it is possible to infer lessons, guide public policy, and ultimately reduce suicide rates.

The *Lancet* study notes: “Most public attention [on suicide] in India has focused on suicide in farmers” [1]. A significant number of ethnographies, case studies, government reports, and newspaper articles claim that the opening of markets and scaling back of state support that followed the liberalization of the Indian economy in the early-1990s led to an “agrarian crisis” and an increase in farmers’ suicides ([6-8]; see Appendix for a summary of literature). So-called “marginal farmers” with landholdings of less that one hectare, who cultivate capital-intensive cash crops that are subject to price fluctuations, such as coffee and cotton, are most likely to have debts that they are unable to pay back, and are, therefore, at greater risk of committing suicide. This research concentrates on states that have some of the highest suicide rates in the country–such as Andhra Pradesh, Kerala, Karnataka and Maharashtra. Thus, when taken as a whole this body of research suggests that inter-state variation in suicide rates can, at least to some extent, be explained by the characteristics of the rural political economy.

Some of Patel et al.’s findings–for example, that suicide rates are twice as high in rural areas compared to urban areas and that ingestion of pesticides accounts for almost half of all suicides–would seem to confirm the farmers’ suicide thesis. Nevertheless, they state: “our findings do not suggest that suicide is any more prevalent in agricultural workers (including farmers) than it is in any other profession” [1]. This finding received international media attention: for example, it was featured in an article on the BBC news website that was titled “Indian farmers and suicide: How big is the problem?” [9]. How can we explain the fact that Patel’s et al. national-level quantitative analysis apparently contradicts such a large number of case studies and anecdotal accounts? There is a tendency to view large-N statistical research as more reliable than narrowly focussed qualitative analyses, particularly in medical sciences. To some extent this assumption is reasonable. As with any case study, these analyses select on the dependent variable. In other words, they attempt to understand farmers’ suicides by focusing on an area that is affected by this phenomenon. It is not possible to generalize from this body of research because it is plausible that there are other areas with similar political and economic conditions that are not affected by farmers’ suicides and therefore not studied. Consequently, it would be easy to conclude that Patel et al.’s study falsifies a widely cited “pseudo fact” established by unreliable case study data and that the issue of farmers’ suicides is merely a “pseudo problem” [10].

The *Lancet* study does not, however, correctly operationalize the mechanism that many case studies identify as linking farmers to increased suicide rates [1]. Patel et al. reach their conclusion by comparing the primary occupations of those people who committed suicide, noting that while agricultural workers accounted for 30% of suicides, non-workers and others (salaried, professional and other jobs) accounted for 33% and 38% respectively.

This is problematic on two main counts. Firstly, it reifies the occupational structure of rural India because in social reality there is not a neat separation between agricultural workers, non-workers, and others. Case studies demonstrate that, before they commit suicide, many struggling farmers undertake wage labour to supplement their meagre income or quit cultivation altogether to begin another occupation [8]. If their income from non-agricultural activities exceeds their income from farming–which is highly likely in a period of agrarian crisis–they would not be classified as a farmer in Patel et al.’s study [1]. Nevertheless, if such an ex-farmer committed suicide, one would have a very strong case for arguing that this should be classified as a “farmer’s suicide”. Secondly, case studies of farmers’ suicides do not argue that the phenomenon accounts for all suicides or that it affects all farmers. Rather, they claim that farmers with certain socioeconomic characteristics–those with marginal landholdings, who cultivate cash crops and are indebted–are at particular risk of committing suicide. Patel et al. do not have the data to test this proposition and are only able to correlate suicide with occupation, as well as other general characteristics such gender, education, geographical region, and marital status [1].

## Methods

This study combines Patel et al.’s state-level suicide rates with other variables to investigate whether the findings identified by narrowly focussed case studies can be generalized to the whole of India. More specifically, we test the hypothesis that suicide rates will be higher in states where there are more marginal farmers, where more cash crops are grown, and where there are more indebted farmers. To the best of our knowledge, which is based on systematic searches of PubMed and Web of the Knowledge, and with the notable exception of Patel et al.’s paper, this is the first study to analyse India-wide quantitative data to investigate whether the findings of qualitative studies of farmers’ suicides are generalizable.

Following Patel et al., we divide India into 18 units: the seventeen largest states, including undivided Bihar, Madhya Pradesh and Uttar Pradesh, as well as a combined unit for the smaller north-eastern states that comprises Sikkim, Arunachal Pradesh, Nagaland, Manipur, Mizoram, Tripura, and Meghalaya.

The dependent variable is the age standardized male suicide mortality rate (suicides per 100,000 per year). Patel et al.’s figures are based on data collected between 2001 and 2003–although they are adjusted to give estimates for 2010. The data comes from a nationally representative mortality survey designed to determine the cause of deaths in 1 · 1 million homes in 6,671 randomly selected small areas. Patel et al.’s estimates (μ = 26.1) are considerably higher than the official and annually enumerated National Crime Records Bureau (NCRB) figures (μ = 15 · 5). This disparity can be explained by the fact that the latter are compiled from local police reports and suicide is illegal–as well as heavily stigmatised–in India [11]. Nevertheless, there is a very strong correlation between the two figures (r = 0 · 950, p < 0 · 001). For our dependent variable we use NCRB figures and adjust for under- or over-reporting using Patel et al.’s estimates.

The proportion of marginal farmers–farming households with a landholding of less than one hectare–was recorded by the Ministry of Agriculture’s quinquennial “Agricultural Census of India” in 2000–01 and 2005–06 [12]. There was only a small difference between the figures in the two surveys, with the proportion of marginal farmers tending to increase slightly (mean = 1 · 5%) over the period. To estimate annual figures we interpolate between the two data points.

To operationalize cash crops, we use the proportion of land that is used to cultivate “non-food crops”, according to Ministry of Agriculture’s annual “Land Use Statistics at a Glance” [13]. This category comprises oil seeds, fibres, dyes, tanning materials, drugs, narcotics and plantations crops, and includes commodities such as cotton and coffee that have been identified by ethnographic research as being associated with farmers’ suicides.

Both the “Agricultural Census” and “Land Use Statistics” are compiled by the Directorate of Economics and Statistics of the Ministry of Agriculture using a combination of land ownership records where they exist (in 87% of India) and household inquiry in the remaining areas (13%)–north-eastern states, West Bengal, Kerala, Orissa. Bihar figures for marginal farmers do not include Jharkhand and northeastern figures do not include Meghalaya.

Data on farmers’ indebtedness has only been collected once, by the National Sample Survey Organization’s “Situation Assessment Survey of Farmers” in 2003 [14]. The sample included 51,770 households in 6,638 villages. The definition of an indebted farming household is: “if it had any loan in cash or kind and its value at the time of transaction was 300 rupees [$5] or more” [14]. While this is a relatively small amount of money by Western standards, we should bear in mind that the Planning Commission recently stated that 25 rupees ($0.4) a day is an “adequate” daily income in rural India [15]. In our time-series analysis we assume that the proportion of indebted farmers is stable for the two years before and after 2003. In reality we would expect some level of variation over this period. Nevertheless, as the political and economic shocks that brought about the agrarian crisis and mass indebtedness occurred in the early- and mid-1990s, we would not expect to see significant changes over this period [7-9].

Table 1 shows the descriptive statistics and Table 2 is a correlation matrix.

**Table 1:** Descriptive statistics of main variables

**Variable**	**N**	**Mean**	**Std. Dev.**	**Min.**	**Max.**
Suicide rate	90	23·5	17·3	2·1	77·0
Marginal farmers %	90	58·9	21·3	12·3	95·5
Cash crops %	90	23·5	15·4	3·2	58·1
Indebted farmers %	90	50·4	15·8	18·1	82·0

**Table 2:** Correlation matrix of main variables

	**1**	**2**	**3**	**4**
1. Suicide rate	1			
2. Marginal farmers %	·310	1		
3. Cash Crops %	·606	-·223	1	
4. Indebted farmers %	·610	-·251	·534	1

In the next section we investigate the relationship between these variables–first using scatter plots and second with linear regressions.

## Results and discussion

First, we report three scatter plots that combine NCRB suicide figures adjusted for under- or over-reporting using Patel et al.’s estimates with each independent variable.

**Figure 1:**
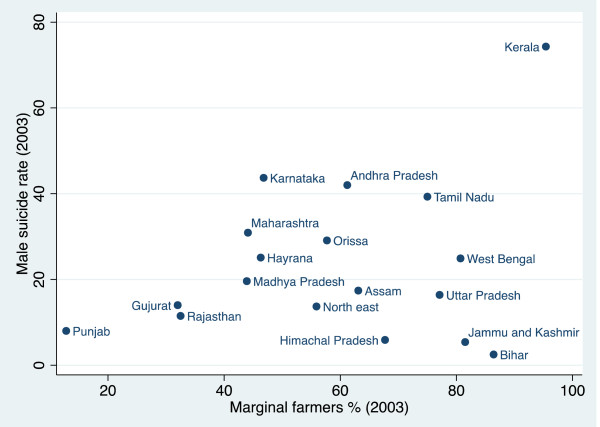
The percentage of marginal farmers and suicide rates.

**Figure 2:**
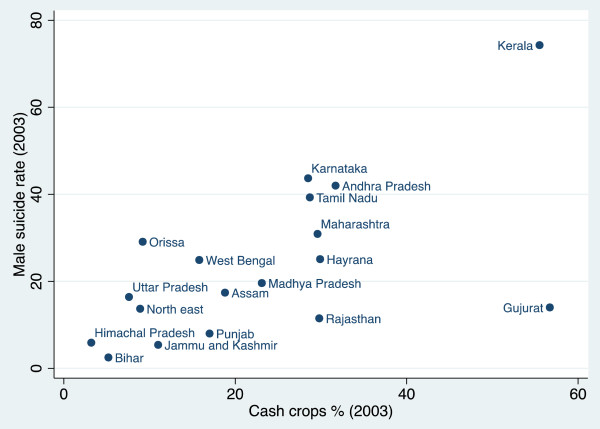
The percentage of crash crops and suicide rates.

**Figure 3:**
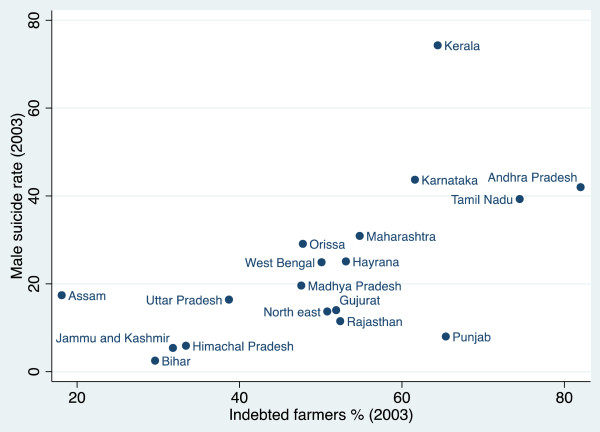
The percentage of indebted farmers and suicide rates.

The relationship between the proportion of marginal farmers and suicide rates is not particularly clear (r = 0 · 241, p = 0 · 378) (Figure 1). The three states with the highest suicide rates–Kerala, Tamil Nadu, and Andhra Pradesh–have some of the highest proportions of marginal farmers and other states–Punjab, Gujarat, and Rajasthan–have both a low proportion of marginal farmers and among the lowest suicide rates. Nevertheless, there is a cluster of states on the bottom right of the scatter plot–Bihar, Jammu and Kashmir, Uttar Pradesh, Himachal Pradesh, the north-eastern states, and Assam–which have a high percentage of marginal farmers but low suicide rates and therefore do not fit into this pattern. It is apparent from Figures 2 and 3 respectively that this cluster of states also has the lowest proportion of both cash crops and indebtedness in India. This suggests that the percentage of marginal farmers is only associated with higher suicide rates in states where farmers are subject to the vulnerability of cash crop cultivation and indebtedness. West Bengal is another outlier, with a relatively high proportion of marginal farmers but a suicide rate only just above the mean. This anomaly might be explained by strength of the Communist Party of India (Marxist) in the state over the past 35 years and, in particular, their unrivalled commitment to improve the hitherto precarious position of marginal farmers [16].

Figure 2 shows there is a clear association between the proportion of cash crops and the suicide rate (r = 0 · 628, p = 0 · 005). The two anomalies are Gujarat and to a lesser extent Rajasthan, which have high levels of cash crops and low suicide rates. Nevertheless, they both have among the lowest proportion of marginal farmers (see Figure 1). Gujarat and Rajasthan’s relatively low suicide rates might be explained by the fact that in these states cash crops tend to be cultivated by farmers with relatively large landholdings and the resources to endure difficult periods without suffering from the same economic problems as marginal farmers.

Figure 3 shows there is a clear association between the percentage of indebted farmers and suicide rates (r = 0 · 729, p = 0 · 018). With a high proportion of indebted farmers but low suicide rate, Punjab, is an outlier. Figure 1 demonstrates that Punjab has by far the lowest proportion of marginal farmers. This indicates that indebtedness is less likely to lead to suicide where farmers have larger landholdings, more resources, and therefore a greater ability to endure difficult periods.

The scatter plots demonstrate that high levels of marginal farmers, cash crops production and indebted farmers are not, on their own, sufficient conditions for high levels of suicide. Nevertheless, there is a clear positive association between the proportion of both cash crops grown and indebted farmers and suicide rates and, while the association between the proportion of marginal farmers and suicide rates is not so clear, we would expect it to become significant when we control for either or both cash crops and indebtedness.

The results of pooled linear regressions using data for the period 2001–05 are shown in Table 3. We report the regression coefficient and in parentheses we specify robust standard errors clustered by state to account for the non-independence of observations from the same state.

**Table 3:** Linear regressions showing the political and economic determinants of farmers’ suicide, 2001-05

	**(1)**	**(2)**	**(3)**	**(4)**	**(5)**	**(6)**
Marginal farmers %	·252			·381*	·402*	·437***
	(·233)			(·123)	(·163)	(·095)
Cash crops %		·683§		·801**		·518*
		(·355)		(·240)		(·209)
Indebted farmers %			·670*		·806**	·549*
			(·214)		(·231)	(·187)
Model N	90	90	90	90	90	90
Model F	1.17	3.70	9.83*	13·97***	6·53*	18·06***
Model adjusted R^2^	·086	·359	·365	·566	·591	·741

*Notes:-* *p < = · 05 (5%), **p < = · 01 (1%), ***p < = · 001 (0.1%), § p = ·071 (7.1%). Constants calculated but not reported.

The results are in line with what we would expect from analysing the scatter plots. The proportion of marginal farmers is not significantly related to suicide rates but the sign of the coefficient is positive (model 1). The significance of the regression model that includes the proportion of cash crops is just above the five per cent level, indicating that there is a positive relationship with suicide rates (model 2). The proportion of indebted farmers displays a significant positive relationship with suicide rates (model 3). The percentage of marginal farmers becomes significant when we add either cash crops or indebted farmers in the regression equation (models 4 and 5). When we include marginal farmers, cash crops, and indebted farmers in the same regression equation all three variables are significant (model 6). We would expect an increase of 0 · 437 in a state’s suicide rate for every one per cent increase in marginal farmers, assuming that all other variables in the model are held constant. A one per cent increase in the percentage of cash crops and indebted farmers would respectively result in a 0 · 518 and 0 · 549 increase in the suicide rate. The adjusted R^2^ for model 6 indicates that 74% of the variability in state-level suicide rates is accounted for by marginal farmers, cash crops, and indebted farmers.

We performed a number of tests on the robustness of model 6. We tested for the possibility that the percentage of marginal farmers, cash crops, and indebted farmers are actually proxies for more general state-level characteristics, such as per capita income [17], poverty [18], or inequality [19] (see Table 4). These variables have been shown to be significantly related to other health outcomes in India [20]. We found that neither per capita income nor percentage of the population below the poverty line are significant when included in the regression equation on their own or alongside the proportion of marginal farmers, cash crops, and indebted farmers (models 7–10). There is a significant positive relationship between Gini coefficient of per capita consumer expenditure and suicide rates: in other words, suicide rates tend to be higher in states that are more unequal (model 11). Nevertheless, the relationship disappears when we control for indebtedness, marginal farmers, and cash crops (model 12). This demonstrates that the proportion of marginal farmers, cash crops, and indebted farmers are better predictors of suicide rates than inequality.

**Table 4:** Linear regressions showing the effects of income, poverty, inequality on farmers’ suicide, 2001-05

	**(7)**	**(8)**	**(9)**	**(10)**	**(11)**	**(12)**
Income	·554	·051				
	(·491)	(·223)				
Poverty			- · 063	·233		
			(·339)	(·133)		
Inequality					·322*	·092
					(.115)	(·073)
Marginal farmers %		·441***		·428***		·429***
		(·099)		(.099)		(·093)
Cash crops %		·514*		·549*		·448§
		(·208)		(·205)		(·215)
Indebted farmers %		·542*		·571*		·395*
		(·189)		(·184)		(·169)
Model N	90	90	90	90	75	75
Model F	1.27	14·83***	·03	16·39***	7.88*	14·12***
Model adjusted R^2^	·038	·738	·010	·757	·361	·735

*Notes:-* *p < = · 05 (5%), **p < = · 01 (1%), ***p < = · 001 (0.1%), § p = ·056 (5.6%). Constants calculated but not reported. For inequality there is no data for Himachal Pradesh, Jammu and Kashmir, and the north eastern states.

We also ran the regressions with various alternative specifications of the dependent variable (see Table 5). First, we used the same method to calculate the suicide rates as in the original model, but used figures for the period 2006 to 2010 (i.e. lagged by five years) (model 13). The model remains similar to the original. Second, we used the original NCRB data, unadjusted for under- or over-reporting as identified by Patel et al. (model 14) [1]. In this model the regression coefficients are lower because, as we noted above, the unadjusted NCRB figures underestimate the magnitude of suicide in India [1]. Apart from the effect size, the results are similar to those we get when using the adjusted suicide rates.

**Table 5:** Linear regressions showing the political and economic determinants of farmers’ suicide with alternative specifications of dependent variables, 2001-05

	**Lagged**	**NCRB**	**All**	**Female**
	**(13)**	**(14)**	**(15)**	**(16)**
Marginal farmers %	·406***	·224**	·327***	·217*
	(·068)	(·068)	(·062)	(·073)
Cash crops %	·449*	·355*	·300*	·077
	(·170)	(·140)	(·128)	(·097)
Indebted farmers %	·617**	·259§	·474***	·403***
	(·179)	(·128)	(·130)	(·090)
Model N	90	90	90	90
Model F	46·93***	10·79***	33·96***	19·16***
Model adjusted R^2^	·800	·630	·733	·596

*Notes:-* *p < = · 05 (5%), **p < = · 01 (1%), ***p < = · 001 (0.1%), §p = ·060 (6%). Constants calculated but not reported.

We then tested whether our results were robust to changes in the gender composition of the dependent variable. In the original models we use male suicide rate. This is because qualitative accounts overwhelmingly characterise farmers’ suicide as a phenomenon that affects male farmers who are generally the head of household and responsible for its economic wellbeing. In the popular imagination female suicide tends to be associated with sati–the act of self-immolation by a widow–or family conflict over dowries [20]. An analysis of NCRB data suggests that, in the period 1997–2006, 85 per cent of farmers’ suicides were by male farmers and that over this period the number of male farmers’ suicides increased at a rate of 3% per annum while female farmers’ suicides remained constant [21]. Nevertheless, it is plausible that farmers’ suicides involving females are understated in NCRB data because, although Indian women often do a large proportion of the agricultural work, they are often not classified as farmers because the land is not registered in their names [22].

The coefficients were smaller when we used total suicide rate rather than male suicide rate (model 15). This supports the idea that farmers’ suicide is a phenomenon that disproportionately affects male farmers. Nevertheless, overall the results were largely unaltered with all three independent variables remaining significant. Model 16, which uses female suicide rate as the dependent variable, is different in several respects to the one that uses male suicide rate (model 6). The percentage of marginal farmers is significant (p = ·009), but the regression coefficient is half the size of the coefficient in model 6: assuming that all other variables in the model are held constant we would expect an increase of 0 · 217 in a state’s female suicide rate for every one per cent increase in marginal farmers, compared to an increase of 0 · 437 in the male suicide rate. The percentage of cash crops is a significant predictor of male suicide rate but is not significant when female suicide rate is the dependent variable. But the percentage of indebted farmers is a significant predictor of both female and male suicide rates. A one per cent increase in the percentage of indebted farmers would result in a 0 · 403 and 0 · 549 increase in the female and male suicide rate respectively. The adjusted R^2^ indicates that model 16 accounts for about 60% of the variability in state-level female suicide rates, compared to 75% for male suicide rates. These results suggest that, contrary to perceived wisdom, the structure of agrarian production does to some extent explain state-level variation in female suicide rates. Nevertheless, it is better at explaining male suicide rates–most probably because men are ultimately responsible for the household’s economic wellbeing in rural India. This finding suggests that qualitative studies of farmers’ suicides should not ignore women. Indeed, more qualitative research is needed to explain why the proportion of marginal farmers and indebted farmers are significant predictors of female suicide but the percentage of cash crops is not.

Table 6 reports two further robustness tests. In model 17 we run the regression without using sandwich estimators for standard errors: that is, standard errors are not clustered by state to account for the non-independence of observations from the same state. The standard errors in this model are, on average, between 2 and 3 times smaller than when we use a sandwich estimator (model 6). This might suggest that the error terms do not have constant variance–i.e., they are heteroskedastic. We therefore undertook a Breusch-Pagan/Cook-Weisberg test (which tests the null hypothesis that the error variances are all equal versus the alternative that the error variances are a multiplicative function of one or more variables). The small and non-significant chi-square value (Χ^2^ = 0 · 05, p = 0 · 83) indicates that heteroskedasticity is not a problem in our analysis.

**Table 6:** Linear regressions showing the political and economic determinants of farmers’ suicide with alternative specifications, 2001-05

	**Without robust SEs**	**Calendar year**
	**(17)**	**(18)**
Marginal farmers %	·437***	·438***
	(·046)	(·095)
Cash crops %	·518***	·518*
	(·072)	(·211)
Indebted farmers %	·549***	·549*
	(·071)	(·189)
Calendar year		- · 087
		(·272)
Model N	90	90
Model F	85.66***	15·25***
Model adjusted R^2^	·741	·738

*Notes:-* *p < = · 05 (5%), **p < = · 01 (1%), ***p < = · 001 (0.1%). Constants calculated but not reported.

Finally, the regression model is more or less unaltered when we include a calendar year variable to control for possible changes over time, such as external shocks to economy (model 18). There were no outliers with standardized residuals of |2|.

These tests increase our confidence in the finding that differences in the structure of agricultural production explain a large amount of inter-state variation in Indian suicide levels. While this analysis is, due to the availability of data, necessarily at a relatively high level of aggregation and only covers a short period of time, our confidence in these findings is considerably increased by the large amount of qualitative data that corroborates our conclusions (see Appendix). There is clearly room for collecting and analysing better quantitative data. More disaggregated or survey data would increase our certainty that the findings are not the result of ecological fallacy, while time-series data would allow us to test whether there was a causal relationship between liberalization and suicide, as argued by qualitative studies and as demonstrated, for example, in post-soviet central and eastern Europe [23].

## Conclusions

This paper investigated whether differences in the structure of agricultural production explain inter-state variation in suicides rates in India. The hypothesis is supported by a large number of qualitative studies, which argue that the liberalization of the agricultural sector in the early-1990s led to an agrarian crisis and that consequently farmers with certain socioeconomic characteristics–cash crops cultivators, with marginal landholdings, and debts–are at particular risk of committing suicide. A recent *Lancet* study, however, contends that there is no quantitative evidence to support this hypothesis. We argue that Patel et al. do not correctly operationalize the mechanism specified by qualitative research on farmers’ suicides. Our analysis combined Patel et al.’s state-level suicide estimates with additional variables to demonstrate that there is a significant and positive relationship between the percentage of marginal farmers and suicide rates, but only when we control for either or both cash crop production and indebted farmers.

Our findings have clear policy implications: they suggest that if the state were able to reduce the proportion of marginal farmers, cash crops, or indebted farmers by one per cent, the suicide rates–suicides per 100,000 per year–would be reduced by 0 · 437, 0 · 518 and 0 · 549 respectively, when all other variables are held constant. Despite more than six decades of trying, the majority of Indian states have been unable to enact meaningful land reforms, largely because of the strength of the rural elite at the local level [16]. Thus, while redistribution of land is a desirable policy prescription, it is perhaps not a realistic one. But even if the size of landholdings are left untouched, our analysis indicates that state interventions to stabilize the price of cash crops and relieve indebted farmers may be effective at reducing suicide rates in India.

## Appendix: summary of literature on farmers’ suicides

Patel et al.’s article only cited one study of farmers’ suicides [1,24]. Nevertheless, there is a large body of research that explores this phenomenon and provides fascinating “thick” data on the micro-dynamics of farmers’ suicides. These studies are predominately published in Indian social sciences journals, most notably *Economic and Political Weekly*. (A significant number of government reports and newspaper articles–the latter most notably by Palagummi Sainath–have also informed our understanding of farmers’ suicides.) This research forms the empirical foundation for the hypothesis that suicide rates will be higher in states where there are more marginal farmers, where a higher proportion of cash crops are grown, and where there are is a greater proportion of indebted farmers.

The vast majority of accounts of farmers’ suicides focus on one of a few relatively small areas of India: the Vidarbha region in eastern Maharashtra [24-28], the plains of Karnataka [29,30], and the Telengana region of northern Andhra Pradesh [31-34], where farmers’ incomes depend on cotton cultivation; and Wyanad district and neighbouring areas in Kerala, where coffee is the major cash crop [35-39].

These studies tend to make very similar assertions regarding the causes of farmers’ suicides. First, it is argued that there is an agrarian crisis in India: “The increasing incidence of farmers’ suicides is symptomatic of a larger crisis, which is much more widespread” [24]. The agrarian crisis is most often related to the liberalization of the Indian economy: “the adoption of the neoliberal model of capitalism by the ruling elite in India since the early 1990s have led to distinct aggregate-level institutional and policy changes related to public investment, input subsidies, organized credit and external trade” [8]. These changes have increased the cost of inputs, while the price of produce has either decreased or become far more volatile. Farmers with small and marginal landholdings, who cultivate cash crops such as coffee and cotton, have been particularly hard hit by these changes.

Cotton cultivation requires relatively large capital expenditure and it is widely argued that these costs have increased dramatically since the liberalization of the economy [8,24-34,40,41]. Due to restrictions put in place by multinational companies, seeds need to be bought every year. Large quantities of fertilizers and pesticides are required, and these have become increasingly expensive due to a reduction in subsidies. In addition, cotton cultivation is very water intensive, but since the early 1990s the amount of public money spent on irrigation has fallen and farmers are increasingly forced to invest in their own systems. In many cases, cotton cultivators must borrow money to pay for these capital outlays and this is particularly true for marginal farmers with very few resources.

Explanations of farmers’ suicides in the coffee growing regions of Kerala are framed in similar terms. But, whereas accounts of suicide among cotton cultivators tend to focus on the increasing costs of inputs, analyses of suicides among coffee farmers concentrate on the decreasing price paid for the produce [8,35-39]. In 2006, for example, the price of coffee was Rs.24 ($0.4) a kilo, whereas it had been more than Rs.130 ($2.2) a kilo a few years previously [8]. This is blamed on the purchasing power of multinational companies who continue to sell the end product at a high price. Falling prices have had a disastrous effect on the livelihoods of 60,000 or so farmers who cultivate coffee on about 70,000 hectares of plantations in Wayanad.

Indebtedness, as one account points out, is the “proverbial last straw” in the causal chain [28]: “The build-up of farmer debt is a direct result of the deepening agrarian crisis, and the wave of farmer suicides is a direct outcome of mounting debt” [8]. A study of the Vidarbha region in Western Maharashtra found that out of 111 cases of farmers’ suicide, 96 (87%) of the families interviewed cited indebtedness as an important reason for their relative’s suicide [24,25]. Debt makes marginal farmers extremely vulnerable to disruptions such as illness or crop failure as a result of extreme weather or pests. Some observers have suggested that the introduction of genetically modified varieties of crops since liberalization has considerably worsened the situation: the cultivation of such crops is “ecologically vulnerable since it is based on monoculture of introduced varieties and on non-sustainable practices of chemically intensive farming” [42].

## Competing interests

The authors declare that they have no competing interests.

## Authors’ contributions

JK compiled the data, designed and undertook the empirical analysis, and drafted the report. LK contributed to the design of the study, interpretation of the findings, and writing up. Both authors read and approved the final manuscript.
